# Integrated Transcriptomic and Metabolomic Analyses Shed Light on the Regulation of Aromatic Amino Acid Biosynthesis in a Novel Albino Tea (*Camellia sinensis*) Mutation

**DOI:** 10.3390/cimb47080644

**Published:** 2025-08-12

**Authors:** Ying Gao, Suimei Li, Xiaojia Zhang, Shuwei Yu, Xinyu Liu, Changbo Yuan, Yuantao Yao, Fan’an Zhang, Lubin Song

**Affiliations:** 1 State Key Laboratory of Nutrient Use and Management, Jinan 250100, China; gying@zju.edu.cn (Y.G.); zhangxiaojia871230@163.com (X.Z.); yswyushuwei@163.com (S.Y.); 2 Shandong Academy of Agricultural Sciences, Jinan 250100, China; xinyuliu0820@163.com (X.L.); 83179270@163.com (C.Y.); ytyao007@163.com (Y.Y.); sdnkcys@163.com (F.Z.); 3 School of Agriculture Science and Technology, Shandong Agriculture and Engineering University, Jinan 250100, China; 17757729659@163.com

**Keywords:** aromatic amino acid, alternative splicing, lncRNAs, *Camellia sinensis*, albino mutation

## Abstract

Off-white or yellowish shoots are common in tea plants (*Camellia sinensis* L.), and such albino variations are often accompanied by metabolic reprogramming, including increased contents of amino acids and lower levels of polyphenols. Nonetheless, the molecular mechanisms that underlie these albino variations remain to be fully clarified. Here, we examined the ultrastructural characteristics of novel, naturally occurring, yellowish mutated tea leaves and performed metabolomic analyses on green and albino leaves and stems. Then, transcriptomic analyses were also conducted on green and albino leaves to investigate the mechanistic basis of the albino variation. As expected, the cells of albino tea leaves contained fewer and smaller chloroplasts with disorganized thylakoids and smaller starch granules. Widely targeted metabolomics analysis revealed 561 differentially abundant metabolites between green and albino leaves and stems, but there was little difference between green and albino stems. Then, RNA sequencing of green and albino leaves revealed downregulation of genes associated with light harvesting and photosynthesis, and integration of the metabolomic and transcriptomic results indicated that biosynthesis of aromatic amino acids (AAAs) was strongly upregulated in albino leaves. To gain additional insight into the molecular basis of the increased AAA levels, Oxford Nanopore long-read sequencing was performed on green and albino leaves, which enabled us to identify differences in long non-coding RNAs (lncRNAs) and alternatively spliced transcripts between green and albino leaves. Interestingly, the amino acid biosynthesis genes arogenate dehydratase/prephenate dehydratase (*ADT*) and serine hydroxymethyltransferase (*SHMT*) were highlighted in the lncRNA and alternative splicing analyses, and the transcription factor genes *PLATZ*, *B3 Os04g0386900*, and *LRR RLK At1g56140* showed significant changes in both expression and alternative splicing in albino leaves. Together, our data suggest that biosynthesis of AAAs might be crucial for albino mutations in tea plants and could be coordinated with the regulation of lncRNAs and alternative splicing. This is a complex regulatory network, and further exploration of the extensive metabolic reprogramming of albino tea leaves will be beneficial.

## 1. Introduction

Tea, made from tender shoots and leaves of *Camellia sinensis* (L.) O. Kuntz, is one of the most popular non-alcoholic beverages worldwide, owing in large part to the unique flavors and health benefits provided by its secondary metabolites, including tea leaf polyphenols, amino acids, caffeine, and volatile compounds [1,2]. The metabolite composition of tea is not only influenced by its genetic background but is also modulated by the growth environment [3].

To adapt to changes in the environment, plants typically adjust their metabolism to avoid damage from abiotic and biotic stresses [4]. Tea plants, which originated in southwestern China and have been cultivated in China for more than 5000 years [5,6], frequently exhibit mutant phenomena under specific environmental and climatic conditions, particularly variations in leaf color such as off-white or yellowish shoots [7]. Both temperature-sensitive and light-sensitive albino tea cultivars, which lack chlorophyll and exhibit aberrant chloroplasts [8,9,10], have been compared to normal green tea leaves, revealing that albino cultivars have higher amino acid contents and lower polyphenol levels, characteristics that are valued for their delicate flavor and economic significance [11,12].

Amino acids contribute to the umami taste of tea and form many of the aroma compounds produced during tea processing. The content and composition of amino acids are therefore key factors in tea quality, and the higher amino acid content of albino tea cultivars is responsible for their good taste [6,7,13,14]. But why are amino acid levels high in albino tea leaves? Researchers have found that a lack of photosynthesis and related metabolism results in deficient carbon metabolism and insufficient nitrogen consumption, leading to the accumulation of free amino acids in tea leaves [15,16,17]. Zheng et al. [18] reported that the accumulation of nitrogen-containing compounds, such as nucleotides and their derivatives, amino acid derivatives, and phenolamides, promoted amino acid synthesis in the albino tea cultivar ‘*Huangjinshuixian*’. Recently, increasing numbers of studies have analyzed the reasons for deficient chloroplasts and amino acid accumulation in albino tea cultivars. Zhang et al. [19] found that DNA methylation dynamics were closely associated with chloroplast structure and function in yellowish leaves of the tea cultivar ‘*Zhonghuang 2′*. In another study, the relative abundance of *CsClpP3m*, an intron-retention variant of the *caseinolytic protease complex* (*Clp*), had a dose-dependent effect on the chlorosis of yellowish tea leaves [20]. In addition, CsMYB42 was shown to promote theanine biosynthesis in albino tea leaves by activating the expression of *glutamine synthase 1c* (*CsGS1c*) [21]. Thus, the reasons for accumulation of free amino acids in albino tea leaves appear to be complex and are yet to be fully clarified.

The aromatic amino acids (AAAs) phenylalanine, tyrosine, and tryptophan serve as the building blocks of proteins and are precursors for other primary and secondary metabolites, including components of photosynthetic electron carriers and biologically active natural products, making them essential for plant growth, development, and resistance [22]. While numerous transcriptome and metabolome studies have been conducted on albino tea leaves from various cultivars, research on the regulatory mechanism of AAA biosynthesis remains limited. Here, we aimed to identify the molecular mechanisms regulating AAA biosynthesis in albino tea mutants using multi-omics approaches. Previous research has demonstrated that alternative splicing and lncRNAs are involved in the perception of various environmental stimuli and the regulation of gene expression during plant growth and development [23,24]. We therefore analyzed alternative splicing and lncRNAs in green and albino leaves in order to better understand the molecular mechanisms that underlie AAA accumulation in a novel albino tea mutation.

## 2. Materials and Methods

### 2.1. Plant Materials

**Figure 1:**
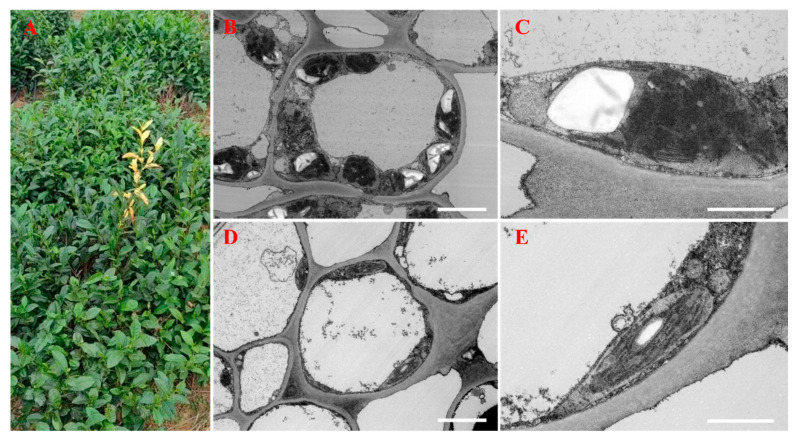
Phenotypes and ultrastructures of green and albino leaves. (**A**) Tea leaf phenotypes. An albino shoot is visible near the center of the image. (**B**) The ultrastructure of a third green leaf. (**C**) The chloroplast ultrastructure of a third green leaf. (**D**) The ultrastructure of a third albino leaf. (**E**) The chloroplast ultrastructure of a third albino leaf. The bars indicate 5 μm in (**B**,**D**) and 2 μm in (**C**,**E**).

A normal tea plant (*Camellia sinensis* cv. Quntizhong) with a yellowish mutation was discovered in October 2023 at a local tea plantation on Lingquan Mountain, Zoucheng City, China (Figure 1A). Green and albino leaves and stems from this plant were collected on October 20, 2023, for use in widely targeted metabolomic analysis. Albino leaves (YLs) and green leaves (GLs) were also collected from the same plant (5 years old) for short-read mRNA sequencing, long-read whole-transcriptome sequencing, electron microscopy, and qRT–PCR. Three replicate samples were collected for each analysis and tissue type.

### 2.2. Ultrastructural Observations of Normal and Albino Leaves

Leaf samples were cut into 1 cm^2^ squares, fixed in a 2.5% glutaraldehyde solution at 4 °C, and post-fixed with 1% OsO4 in a 0.1 M phosphate buffer (pH 7.4) for 7 h at room temperature. The fixed samples were dehydrated and infiltrated as described by Li et al. [6]. Ultrathin sections were obtained with a Leica UC7 ultramicrotome (Leica, Schott, Germany), stained with 2% uranium acetate and 2.6% lead citrate for 5–10 min each, and observed by TEM (model: H-7800, Hitachi, Tokyo, Japan).

### 2.3. Widely Targeted Metabolite Analysis

After vacuum freeze-drying, a 50 mg sample was mixed with 1000 μ L of an extraction solution (methanol/acetonitrile/water volume ratio = 2:2:1). The mixture was treated with a 45 Hz grinder for 10 min, sonicated for 10 min (in an ice water bath), and remained stationary for one hour at −20 °C. Then, the sample was centrifuged at 4 °C and 12,000 rpm for 15 min. Next, 500 μL of the supernatant was moved into an EP tube and dried by a vacuum concentrator. Then, 160 μL of an extraction solution (acetonitrile/water volume ratio: 1:1) was added to the dried metabolites for reconstitution, followed by vortexing for 30 s and sonicating in an ice water bath for 10 min. Then, the sample was centrifuged at 4 °C and 12,000 rpm for 15 min, and 120 μL of the supernatant was carefully taken out and transferred into a 2 mL injection bottle. Next, 10 μL were taken from each sample and mixed to form a QC sample for machine testing by Beijing Biomarker Biotechnology (Beijing, China) using a UPLC-ESI-MS/MS system (UPLC: Waters Acquity I-Class PLUS; MS: Applied Biosystems QTRAP 6500+), with three replicates per sample type [25].

A Waters HSS-T3 UPLC column (1.8 µm, 2.1 mm × 100 mm) was used. The mobile phase of solvent A was pure water with 5 mM Ammonium acetate and 0.1% formic acid, and solvent B was acetonitrile with 0.1% formic acid. The gradient program was as follows: An initial composition of 98% A and 2% B was maintained for 1.5 min. The phase-A concentration decreased to 50% and the phase-B concentration increased to 50% over the next 5.0 min. Then, the phase-A concentration decreased to 2% and the phase-B concentration increased to 98% over another 9.0 min, and a composition of 2% A and 98% B was maintained for 1 min. Subsequently, the concentrations of phases A and B were sharply shifted back to 98% A and 2% B within 1 min and maintained for 3 min. The detection parameters were set as follows: a flow velocity of 0.35 mL/minute, a column oven temperature of 50 °C, and an injection volume of 2 uL. The effluent was alternatively connected to an ESI–triple-quadrupole–linear ion trap (QTRAP)-MS system [26].

Metabolite peaks were normalized based on the total peak area. Then, the fold changes and significant differences were analyzed via Student’s t-test. Principal component analysis (PCA) was conducted by the package prcomp R (v3.6.1). VIP values were calculated via multiple cross-validation. The criteria of fold change > 1, *p* < 0.05, and VIP > 1 were used to identify differentially abundant metabolites. The Kyoto Encyclopedia of Genes and Genomes (KEGG) compound database (http://www.kegg.jp/kegg/compound/) (accessed on 23 October 2023) was used for annotation of metabolites. Then, the annotated metabolites were mapped to the KEGG pathway database. Lastly, the analysis of KEGG pathway enrichment was analyzed by a hypergeometric test [27].

### 2.4. Illumina Transcriptome Sequencing and DEG Identification

RNA extraction from three replicate samples of GLs and YLs was performed via a Trelief RNAprep Pure Plant Plus Kit (Beijing Tsingke Biotechnology Co., Ltd., Beijing, China) according to the manufacturer’s instructions. A Nanodrop 8000 spectrophotometer (Thermo Fisher, Waltham, MA, USA), a Qubit 2.0 fluorometer (Thermo Fisher, Waltham, MA, USA), and an Agilent 2100 bioanalyzer (Agilent Technologies, Santa Clara, CA, USA) were used to detect the purity, concentration, and integrity of the RNA samples.

After library construction, quality control, and quantification, the RNA libraries were sequenced by Beijing Biomarker Biotechnology (Beijing, China) on an Illumina NovaSeq 6000 system in PE150 mode. The quality of the raw sequencing data was controlled on the basis of Q20 scores and the GC content distribution. After 3ʹ adaptor trimming and removal of low-quality reads with cutadapt software (v1.9.3), high-quality clean reads were aligned to the reference genome (CSS_ChrLev_20200506_Genome.fas.gz) (http://tpia.teaplants.cn/download.html) (accessed on 21 October 2023) [28,29] using HISAT2 (v2.0.4) [30]. Gene expression levels were quantified using featureCounts, and FPKM values (fragments per kilobase per million mapped reads) were computed for each gene. DESeq2 (v1.30.1) was used to identify DEGs between the green and albino leaves (FDR-adjusted *p* < 0.05 and |log2(fold change)| > 1), with the Benjamini–Hochberg method used to control the false discovery rate (FDR) [31]. GO term and KEGG pathway enrichment analyses were performed on the identified differentially expressed genes (DEGs) via the ClusterProfiler R package (v4.4.4).

### 2.5. ONT Whole-Transcriptome Sequencing and Analysis of Alternative Splicing and lncRNAs

To assemble all transcripts and identify isoforms, full-length transcriptome sequencing of GL and YL samples was performed by Beijing Biomarker Biotechnology (Beijing, China) using the Oxford Nanopore Technologies platform. First, 1 ug of total RNA was prepared for cDNA libraries using a cDNA-PCR Sequencing Kit (SQK-LSK110+EXP-PCB096) protocol provided by Oxford Nanopore Technologies (ONT) [32]. The resulting raw sequences were filtered to remove low-quality reads (length < 500 bp, Q score < 6) and ribosomal RNA sequences, and full-length non-chimeric sequences were identified as those with primers at both ends. The full-length sequences were polished using Pinfish software (v0.1.0) to obtain consensus sequences. The final transcript sequences were used directly for analyses of alternative splicing and lncRNAs.

ASTALAVISTA (http://genome.imim.es/astalavista) (accessed on 23 October 2023) [33] was used to identify alternatively spliced transcripts in each sample, including those arising from exon skipping, alternative 3′ splice sites, alternative exons, alternative 5′ splice sites, and intron retention. Several widely used methods were used to determine the coding potential and predict lncRNAs among the novel transcripts: the Coding Potential Calculator (CPC) [34], the Coding-Non-Coding Index (CNCI) [35], the Coding-Potential Assessment Tool (CPAT) [36], and Pfam [37] protein domain analysis. lncRNA target genes were predicted on the basis of their locations relative to lncRNAs and by analysis of complementary base pairing using LncTar (http://www.cuilab.cn/lnctar) (accessed on 24 October 2023) [38].

### 2.6. qRT–PCR

The relative expression levels of genes were identified by qRT–PCR (Supplemental Table S5) on a CFX384 Touch Real-time PCR Detection System (Bio-Rad Laboratories, Hercules, CA, USA) with ChamQ SYBR qPCR Master Mix (Nanjing Vazyme Biotech, Nanjing, China). PCR primers were designed using Primer-BLAST (www.ncbi.nlm.nih.gov/tools/primer-blast/) (accessed on 6 November 2024) with default parameters and synthesized by GenScript Biotech (Nanjing, China). The PCR cycling conditions were as follows: 95 °C for 30 s, then 40 cycles at 95 °C for 10 s and 60 °C for 30 s. Reaction specificity was ensured by analysis of the melting curves. Relative gene expression levels were calculated using the 2^−ΔΔCt^ method with β-actin as the reference gene [39]. The qRT–PCR analyses were performed in triplicate.

### 2.7. Statistical Analysis

All experiments were performed with at least three independent replicates (*n* ≥ 3), except for the ONT whole-transcriptome sequencing. Significant differences were identified using Duncan’s multiple range test (*p* < 0.05) performed in SAS Version 9.0 (SAS Institute, Cary, NC, USA).

## 3. Results

### 3.1. Ultrastructural Characteristics of Normal and Albino Leaves

Differences in the ultrastructures of normal and albino leaves (Figure 1A) were observed by transmission electron microscopy (TEM). Chloroplasts from green leaves displayed a typical, well-stacked thylakoid membrane system with large starch granules (Figure 1B,C). By contrast, albino leaves contained fewer and smaller chloroplasts with disorganized thylakoids and small starch granules that were located in the vicinity of the cell membrane (Figure 1D,E). Overall, the mitochondria and chloroplasts in albino leaves appear to be smaller and thinner than those in normal cells.

### 3.2. Differentially Abundant Metabolites in Normal and Albino Leaves and Stems

**Figure 2:**
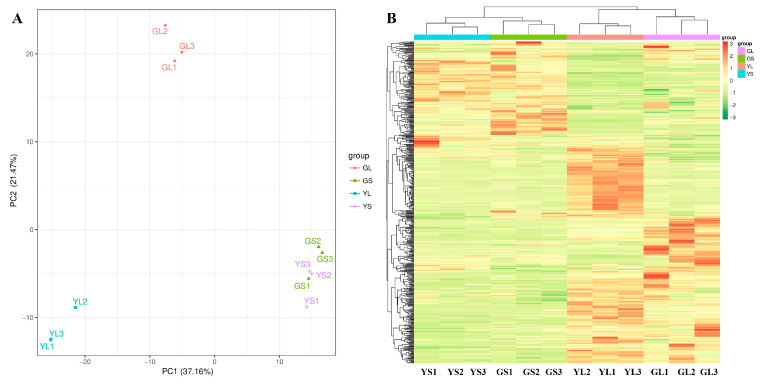
PCA (**A**) and clustering analysis (**B**) of differential metabolites in albino and normal leaves (GLs and YLs) and stems (GSs and YSs).

Widely targeted metabolomics was used to identify differentially abundant metabolites in normal and albino leaves and stems. A total of 561 differentially abundant metabolites were identified between the normal and albino leaves and stems (VIP ≥ 1, *p* < 0.05) (Figure 2B), 376 of which were successfully mapped to the KEGG pathway database. Marked differences in metabolite composition between the normal and albino leaves and between the leaves and stems were observed via PCA (Figure 2A) and hierarchical clustering analysis (Figure 2B), but there were few differences between the albino stems and normal stems. There were 229 differentially abundant metabolites between the normal and albino leaves, 401 differentially abundant metabolites between the albino leaves and stems, and 166 differentially abundant metabolites between the normal leaves and stems, while there were only 95 differentially abundant metabolites between the albino stems and normal stems (Figure 2B).

**Figure 3:**
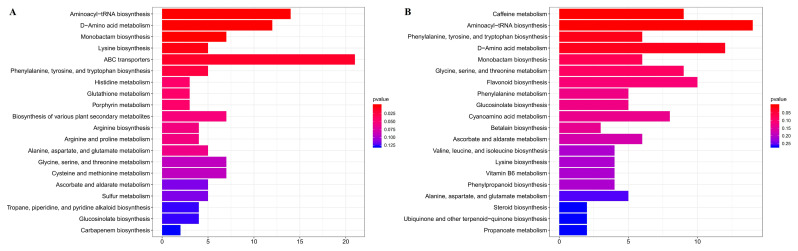
KEGG enrichment analyses of differentially abundant metabolites. (**A**) Normal leaves (GLs) versus albino leaves (YLs). (**B**) Albino leaves (YLs) versus albino stems (YSs). The top twenty enriched pathways for each comparison are shown.

KEGG enrichment analyses of differentially abundant metabolites (Figure 3) ere performed between normal leaves (GLs) and albino leaves (YLs) and between albino leaves (YLs) and albino stems (YSs). A number of KEGG pathways, many associated with amino acid metabolism, were enriched in both comparisons. These included “aminoacyl-tRNA biosynthesis”, “D-amino acid metabolism”, “phenylalanine, tyrosine and tryptophan biosynthesis”, “monobactam biosynthesis”, “lysine biosynthesis”, “glycine, serine and threonine metabolism”, and “alanine, aspartate and glutamate metabolism”, consistent with substantial alterations in the amino acid metabolism of albino leaves. Meanwhile, the changes in L-lysine, L-tryptophan, L-serine, L-threonine, L-tyrosine, and L-phenylalanine were all above 2 folds between the normal leaves (GLs) and albino leaves (YLs) and between the albino leaves (YLs) and albino stems (YSs).

### 3.3. RNA Sequencing, Reference Genome Alignment, and GO Analysis of DEGs

Transcriptome sequencing and analysis of replicated GL and YL samples were performed to investigate the reasons for the marked differences in metabolite composition between the normal and albino leaves. We obtained 38.78 Gb of clean Illumina short-read data from six cDNA libraries (Q20 > 97% and Q30 > 93%), with an average of 6.46 Gb per sample (Supplemental Table S1). The clean reads were mapped to the C. sinensis reference genome using HISAT2, and the proportion of mapped reads was approximately 87% for each sample (Supplemental Table S2), confirming the high quality of the data.

**Figure 4:**
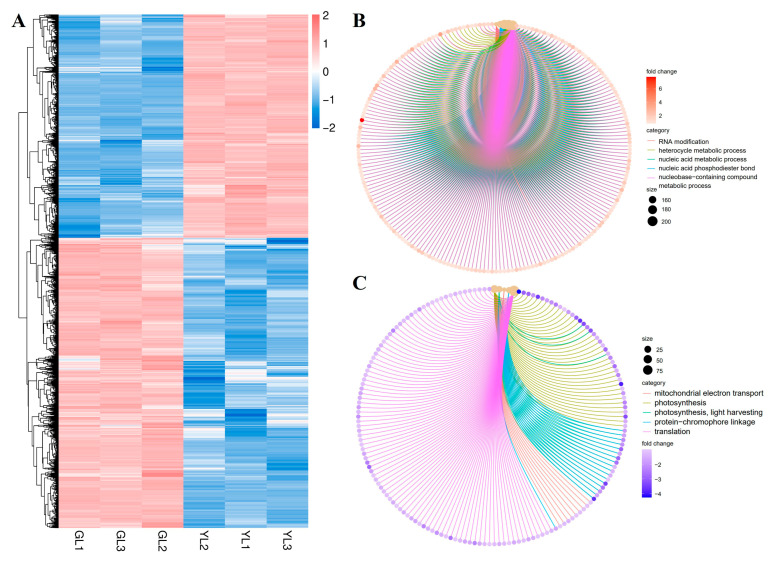
Hierarchical clustering and GO enrichment analysis of DEGs from normal and albino leaves. (**A**) Heatmap of DEGs in normal leaves (GLs) versus albino leaves (YLs). (**B**) GO enrichment analysis of DEGs upregulated in YLs versus GLs. (**C**) GO enrichment analysis of DEGs downregulated in YLs versus GLs.

A total of 9637 DEGs (|fold change| ≥ 2, FDR < 0.01) were identified between the GL and YL samples: 4192 were upregulated and 5445 were downregulated in YLs versus GLs (Figure 4A). Analysis of gene ontology (GO) biological process terms revealed that the upregulated genes were enriched in “RNA modification”, “heterocycle metabolic process”, “nucleic acid metabolic process”, “nucleic acid phosphodiester bond hydrolysis”, and “nucleobase-containing compound metabolic process” (Figure 4B), whereas the downregulated genes were enriched in “mitochondrial electron transport, ubiquinol to cytochrome”, “photosynthesis”, “photosynthesis, light harvesting”, “protein-chromophore linkage”, and “translation” (Figure 4C). These results suggest that nucleic acid metabolism and photosynthesis were markedly altered in the albino leaves at the transcriptional level.

### 3.4. Integrated KEGG Analysis of Metabolites and DEGs in Normal and Albino Leaves

**Figure 5:**
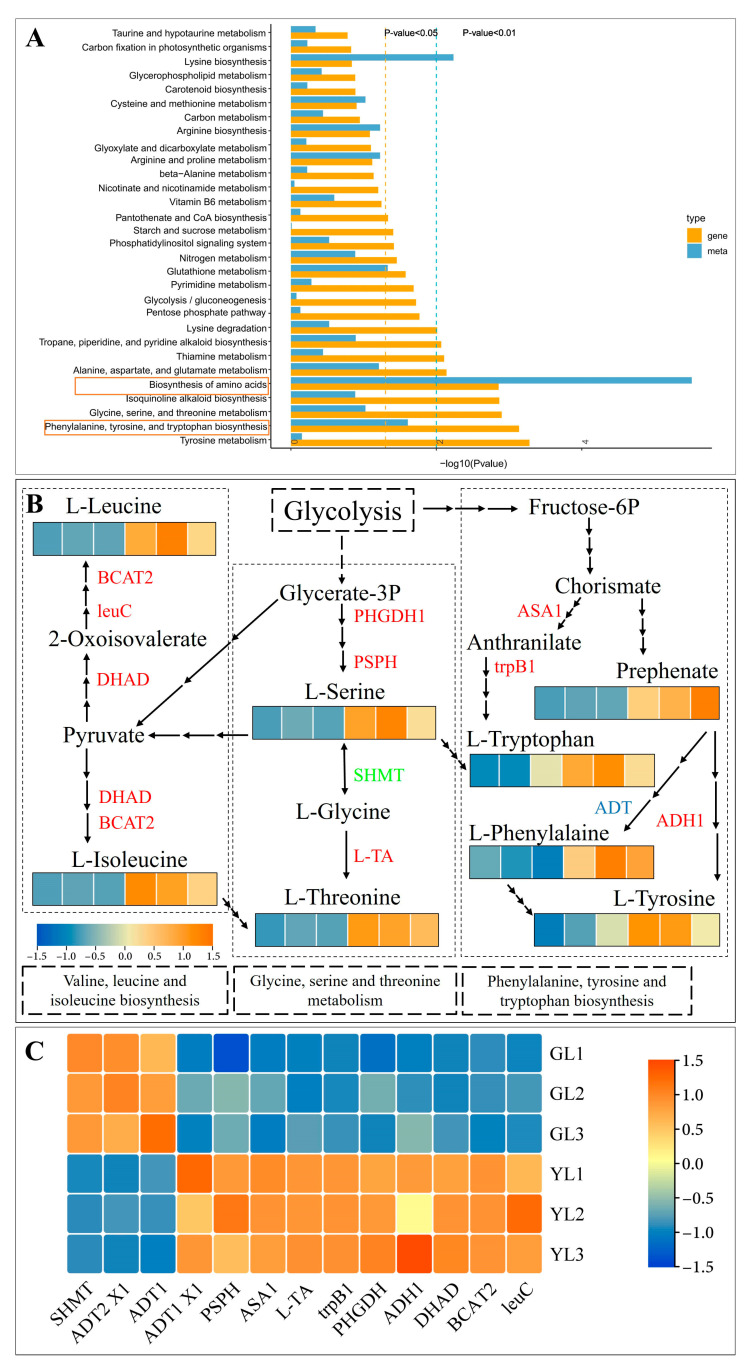
Integrated analysis of differentially abundant metabolites and DEGs in normal leaves (GLs) and albino leaves (YLs). (**A**) Integrated KEGG enrichment analysis, with yellow boxes highlighting two KEGG pathways that were significantly enriched in both the differentially abundant metabolites and DEGs. The blue and yellow dotted lines represent the significance of enrichment (*p* < 0.05 and *p* < 0.01, respectively). (**B**) Integrated analysis of key genes and metabolites associated with the enriched KEGG pathway “biosynthesis of amino acids”. Heatmaps show the abundance of key metabolites in this pathway, and the red, green, and blue fonts indicate genes that were upregulated, downregulated, and both up- and downregulated in YLs versus GLs, respectively. (**C**) The relative expression of key genes in the “biosynthesis of amino acids” pathway as measured by qRT–PCR. Each heatmap contains 6 cells, representing three biological samples from GLs and YLs. BCAT2: branched-chain amino acid aminotransferase; leuC: 3-isopropylmalate dehydratase large subunit; DHAD: dihydroxy-acid dehydratase; PHGDH1: D-3-phosphoglycerate dehydrogenase 1; PSPH: phosphoserine phosphatase; SHMT: serine hydroxymethyltransferase; L-TA: low-specificity L-threonine aldolase 1; ASA1: anthranilate synthase alpha subunit 1; trpB1: tryptophan synthase beta chain 1; ADT1: arogenate dehydratase/prephenate dehydratase 1; ADT1 X1: arogenate dehydratase/prephenate dehydratase 1 isoform X1; ADH1: arogenate dehydrogenase 1.

The enriched KEGG pathways of the differentially abundant metabolites were compared to those of the DEGs, and it was found that “biosynthesis of amino acids” was strongly and significantly enriched in both (Figure 5A), with 136 DEGs and 20 differentially abundant metabolites. The related KEGG pathway “phenylalanine, tyrosine and tryptophan biosynthesis” was also significantly enriched in both (Figure 5A). We therefore focused our attention on genes and metabolites associated with “phenylalanine, tyrosine and tryptophan biosynthesis”, as well as the closely related pathways “glycine, serine and threonine metabolism” and “valine, leucine and isoleucine biosynthesis” (Figure 5B). The contents of L-phenylalanine, L-tryptophan, L-tyrosine, L-serine, L-threonine, L-leucine, and L-isoleucine were all clearly increased in the albino leaves. Consistent with this result, numerous genes associated with the metabolism of these amino acids were differentially expressed in the albino leaves. These included anthranilate synthase alpha subunit 1 (*ASA1*), tryptophan synthase beta chain 1 (*trpB1*), arogenate dehydrogenase 1 (*ADH1*), arogenate dehydratase/prephenate dehydratase 1 isoform X1 (*ADT1 X1*), D-3-phosphoglycerate dehydrogenase 1 (*PHGDH1*), phosphoserine phosphatase (*PSPH*), low-specificity L-threonine aldolase 1 (*L-TA*), dihydroxy-acid dehydratase (*DHAD*), 3-isopropylmalate dehydratase large subunit (*leuC*), and branched-chain amino acid aminotransferase 2 (*BCAT2*), all of which were upregulated, and arogenate dehydratase/prephenate dehydratase 1/2 isoform X1 (*ADT1/ADT2 X1*) and serine hydroxymethyltransferase (*SHMT*), which were downregulated. Downregulation of SHMT would be expected to inhibit the interconversion of L-serine and L-glycine and to promote the accumulation of L-tryptophan, an aromatic amino acid (AAA). Indeed, the L-tryptophan content was higher in the albino leaves, as were those of the other two AAAs, L-phenylalanine and L-tyrosine. Thus, the albino leaf phenotype appeared to be associated with higher levels of AAAs.

### 3.5. Analysis of lncRNAs and Their Targeted DEGs Involved in AAA Biosynthesis

**Figure 6:**
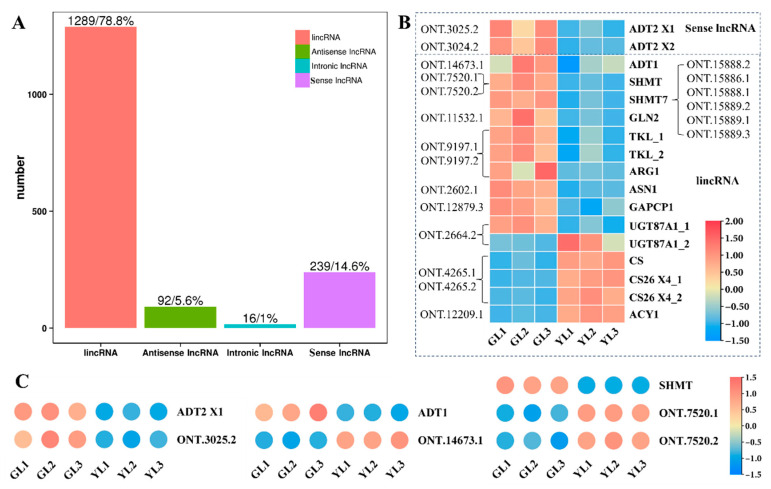
Analysis of lncRNAs and their predicted DEG targets associated with the KEGG pathway “biosynthesis of amino acids”. (**A**) The numbers of different types of lncRNA obtained in C. sinensis leaves. (**B**) lncRNAs and their predicted DEG targets associated with the “biosynthesis of amino acids” KEGG pathway. (**C**) The relative expression of *ADT2 X1*, *ADT1*, *SHMT*, and their predicted cognate lncRNAs as measured by qRT–PCR.

To further explore the molecular changes that underlie the albino variation, Oxford Nanopore long-read sequencing of whole GL and YL transcriptomes was performed in order to predict long non-coding RNAs (lncRNAs) and their potential targets. An average of 6.07 Gb was obtained per sample (Supplemental Table S3), and the proportion of full-length reads exceeded 89.7% for each sample (Supplemental Table S4). We identified a total of 1636 candidate lncRNAs, including 92 anti-sense lncRNAs, 16 intronic lncRNAs, 1289 long intergenic non-coding RNAs (lincRNAs), and 239 sense lncRNAs (Figure 6A). We then predicted their target genes and found that 17 DEGs associated with the “biosynthesis of amino acids” KEGG pathway were potentially targeted by 20 lncRNAs (Figure 6B). Three predicted targets were ADT genes (*ADT1*, *ADT1 X1*, and *ADT2 X1*) that were also associated with the “phenylalanine, tyrosine and tryptophan biosynthesis” pathway, and two were *SHMT* genes (*SHMT* and *SHMT7*) also associated with the “glycine, serine and threonine metabolism” pathway, consistent with the integrated KEGG enrichment analysis above. Among the 20 lncRNAs predicted to target amino acid-related DEGs, ONT.3024.2 and ONT.3025.2 were sense lncRNAs and the remainder were lincRNAs. A quantitative real-time polymerase chain reaction (qRT–PCR) revealed that the expression profile of ONT.3025.2 was similar to that of *ADT2 X2*, whereas the expression levels of *ADT1* and *SHMT* appeared to be negatively correlated with those of their predicted cognate lncRNAs (Figure 6C). All of these results suggest that lncRNAs may have a role in regulating changes in AAA biosynthesis in albino leaves.

### 3.6. Analysis of Alternatively Spliced Transcription Factor Genes

**Figure 7:**
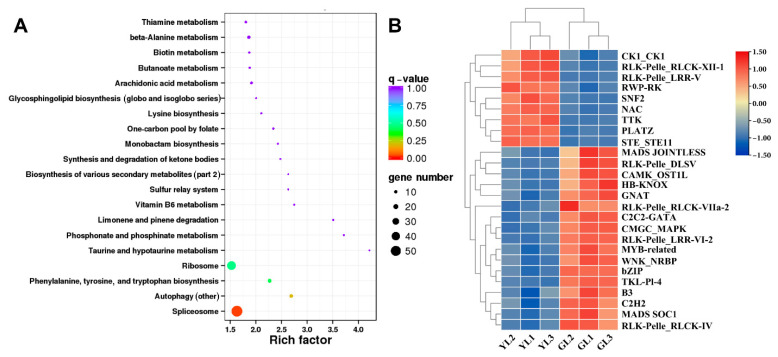
Analysis of alternatively spliced transcription factor genes. (**A**) Top twenty enriched KEGG pathways of alternatively spliced transcription factor genes. (**B**) Expression heatmap of alternatively spliced transcription factor genes.

ONT whole-transcriptome sequencing data were used to examine alternative splicing in the normal and albino leaves. A total of 3503 alternative splicing events involving 2251 genes were identified through alternative splicing analysis. Genes with alternatively spliced transcripts were enriched in KEGG pathways such as “spliceosome”, “phenylalanine, tyrosine, and tryptophan biosynthesis”, and “ribosome” (Figure 7A), suggesting that alternative splicing may influence differences in transcription, translation, and amino acid metabolism in albino leaves. Interestingly, alternative splicing occurred in the phenylalanine biosynthesis gene *ADT1 X1* and therefore may have been associated with the upregulated *ADT1 X1* expression and increased phenylalanine content of the albino leaves (Figure 5B,C).

**Figure 8:**
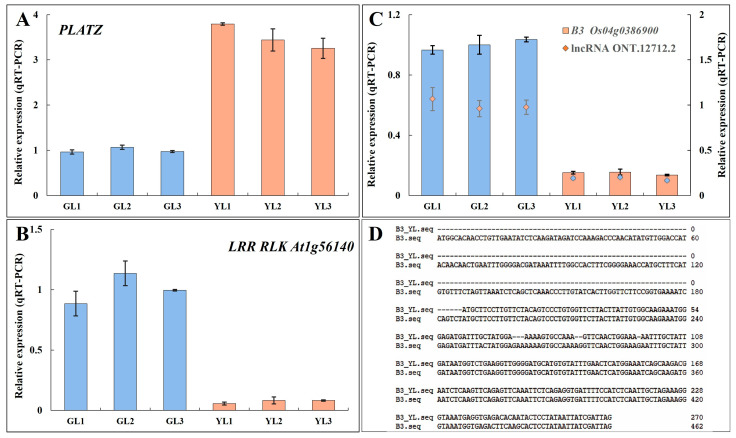
The expression levels and alternative sequences of three transcription factors that exhibited alternative splicing. (**A**,**B**) The relative expression levels of *PLATZ* (**A**) and *LRR RLK At1g56140* (**B**) as measured by qRT–PCR. (**C**) The relative expression levels of *B3 Os04g0386900* and the lncRNA ONT.12712.2. (**D**) The alternatively spliced sequence of *B3 Os04g0386900* in the YLs.

Transcription factor prediction analysis of DEGs with alternative splicing identified a total of 50 transcription factors. Among them, 26 were also differentially expressed in the transcriptome (Figure 7B). Genes encoding the PLant AT-rich protein and Zinc-binding protein transcription factor (*PLATZ*), the B3 domain-containing protein Os04g0386900 (*B3 Os04g0386900*), and the LRR receptor-like serine/threonine protein kinase At1g56140 (*LRR RLK At1g56140*) exhibited significant differential expression (|fold change| ≥ 5) between the albino and normal leaves in both Illumina-based and ONT-based transcriptomes. *PLATZ* was upregulated in albino leaves, whereas *B3 Os04g0386900* and *LRR RLK At1g56140* were downregulated. Moreover, B3 Os04g0386900 was predicted to be spliced by lncRNA ONT.12712.2, leading to a shorter open reading frame at the 5′ end and a lower expression level. The expression levels of these genes were verified by qRT–PCR (Figure 8), confirming that *PLATZ* was significantly upregulated in the albino leaves, although whether its expression is related to increased AAA biosynthesis will require further research.

## 4. Discussion

Recent studies have shown that in addition to low chlorophyll contents, the contents of amino acids are higher in albino tea leaves, especially L-theanine [40,41]. In the present study, we analyzed a novel albino tea mutation using metabolomics and short- and long-read RNA sequencing to gain a comprehensive picture of the molecular reprogramming associated with its leaf color variation. We found that many of the differentially abundant metabolites in albino mutant tea leaves were involved in amino acid metabolism, especially the biosynthesis of AAAs. On the other hand, DEGs between green and albino leaves were mainly involved in photosynthesis and light harvesting, suggesting that the accumulation of AAAs may be closely related to changes in carbon metabolism in albino leaves.

Integrated analysis revealed a clear association between increased AAA biosynthesis and leaf color variation. AAAs participate in the formation of various primary and specialized metabolites, among which the key cell wall polymer lignin is the most abundant. These metabolites also include flavonoids, isoflavonoids, tannins, and volatiles [22]. All of these compounds are important for plant growth, development, and environmental responses [42,43,44]. Due to the imbalance between carbon and nitrogen metabolism, albino tea plants are expected to adopt other approaches to deal with strong photoinhibition and reactive oxygen species accumulation [45,46], and a variety of AAA derivatives may be needed for stress protection in albino leaves. Therefore, the higher AAA contents observed here may help albino tea leaves to resist environmental stress.

Regulation of aromatic amino acid biosynthesis involves a complex transcriptional network. During the final step of phenylalanine biosynthesis, ADT enzymes perform decarboxylation/dehydration of arogenate to produce phenylalanine [47,48]. Here, expression of three ADT genes (*ADT1*, *ADT2*, and *ADT6*) was detected in the sampled tea tissues, and expression of individual *ADT1* and *ADT2* isoforms (*ADT1 X1*, *ADT2 X1,* and *ADT2 X2*) was also observed. The expression of *ADT1*, *ADT2 X1*, and *ADT2 X2* appeared to be correlated with that of specific cognate lncRNAs. The expression of *ADT1* might be trans-regulated by lncRNA ONT.14673.1, while the expression of *ADT2 X1* might be cis-regulated by lncRNA ONT.3025.2. Moreover, alternative splicing appeared to occur in *ADT1 X1*, which was the only clearly upregulated *ADT* gene in the albino leaves and may have contributed to their phenylalanine accumulation. *SHMT*, which participates in the serine–glycine–one-carbon metabolic network [49], was downregulated in the albino leaves and showed an expression pattern opposite to that of its predicted cognate lncRNAs ONT.7520.1 and lncRNA ONT.7520.2. SHMT activity has been detected in chloroplasts in pea, barley, and Arabidopsis, and AtSHMT3 could catalyze the transfer of a hydroxymethyl group from serine to H4PteGlu6 in plastids [50]. In rice, leaves of an osshmt1 transgenic line lost photosynthetic capacity in the electron transport chain during photochemical reactions and CO_2_ assimilation [51]. In cotton, light-responsive elements accounted for the majority of the cis-elements in the *GhSHMT* promoter, and downregulation of *GhSHMT* was accompanied by more curled and yellow leaves, a reduced chlorophyll content, and a noticeable decrease in the net photosynthetic rate [52]. Therefore, it is possible that downregulation of *SHMT* and alternative splicing of *ADT XI* may be responsible for the accumulation of AAAs, which might be an alternative means of sustaining plant growth, development, and stress resistance for albino leaves.

Regulation of transcription factors is also essential for the amino acid biosynthetic network. We found that three transcription factors (*LRR RLK At1g56140*, *B3 Os04g0386900*, and *PLATZ*) all underwent alternative splicing in albino tea leaves, accompanied by significant changes in the expression levels. Only *PLATZ* was upregulated, suggesting that it might be important for the albino leaf variation. In plants, B3 TFs are mainly involved in embryonic development and seed development [53]. LRR RLKs could sense and transduce extracellular signals, which are involved in various biological processes [54]. The *PLATZ* transcription factor family is specific to photosynthetic eukaryotes and participates in leaf and/or seed development and the regulation of meristematic activity [55,56]. In Arabidopsis, the *PLATZ* gene *ORE15* enhanced leaf growth by increasing the rate and duration of cell proliferation during early leaf development and suppressed leaf senescence at a later stage [57]. In poplar, *PtrPLATZ14* was shown to regulate the cell size control genes *PtrGRF/GIF* and *PtrTCP* to influence poplar leaf development [58]. Therefore, the alternatively spliced transcription factor *PLATZ* observed here might play a role in the growth and development of albino leaves, although further research is needed to determine whether it is associated with AAA synthesis.

## 5. Conclusions

In this study, the changes in metabolite composition in leaves were much greater than those in stems. And GO analysis of DEGs in leaves showed nucleic acid metabolism and photosynthesis were markedly enriched. These findings illustrate that photosynthetic metabolism is hindered in albino leaves and that maintaining growth and development necessitates a readjustment of metabolism and gene expression. One example of such a readjustment may be the changes in amino acid-related gene expression and the increased accumulation of AAAs observed here in albino tea leaves. In particular, the AAA-related genes *SHMT* and *ADT* appeared to be coordinately regulated by lncRNAs and alternative splicing. The transcription factor genes *LRR RLK At1g56140*, *B3 Os04g0386900*, and *PLATZ* also appeared to be regulated by alternative splicing and lncRNAs in albino leaves, leading to significant changes in their expression. Although these specific regulatory relationships require further experimental confirmation, our results provide evidence of a complex regulatory network for AAA biosynthesis in albino tea leaves, providing new insight into the molecular mechanisms that underlie albino variations.

## Data Availability

The short and long RNA-Seq data presented in this study were deposited in the NCBI repository with accession number SAMN46042864. The other datasets generated and/or analyzed during the current study are also available from the corresponding author upon reasonable request.

## Supplementary Materials

The following supporting information can be downloaded at https://www.mdpi.com/article/10.3390/cimb47080644/s1.
