# Supplementary material for: Integrated Transcriptomic and Metabolomic Analyses Shed Light on the Regulation of Aromatic Amino Acid Biosynthesis in a Novel Albino Tea (Camellia sinensis) Mutation
Source: Curr Issues Mol Biol. 2025 Aug 12;47(8):644. doi: 10.3390/cimb47080644 (PMC12384648; doi:10.3390/cimb47080644)
Supplement: Supplementary file 1 [file cimb-47-00644-s001.zip › cimb-3749051-supplementary.pdf]

Table S1. Statistical analysis of sequencing data.

Sample	Clean reads	Clean bases	GC (%)	Q20 (%)	Q30 (%)
GL1	22,823,240	6,833,098,456	44.62	97.6	93.81
GL2	21,057,630	6,305,848,089	44.79	97.47	93.52
GL3	23,420,006	7,009,862,851	44.57	97.5	93.41
YL1	21,186,229	6,339,175,259	44.22	97.87	94.11
YL2	20,589,569	6,160,072,559	44.17	97.48	93.1
YL3	20,478,836	6,127,225,635	44.23	97.67	93.64

Note: Sample: sample name; clean reads: counts of clean PE reads; clean bases: total base number of clean data; GC content: percentage of G and C in clean data; Q20: percentage of bases with Q-scores of no less than Q20; Q30: percentage of bases with Q-scores of no less than Q30.

Table S2. Statistical analysis of sequence alignment with selected reference genome.

Sample	Total Reads	Mapped Reads	Unique Mapped Reads	Multiple Mapped Reads	Reads Map to '+'	Reads Map to '-'
GL1	45,646,480	39,904,841 (87.42%)	34,315,263 (75.18%)	5,589,578 (12.25%)	23,918,117 (52.40%)	23,831,679 (52.21%)
GL2	42,115,260	36,815,068 (87.42%)	31,518,786 (74.84%)	5,296,282 (12.58%)	22,316,875 (52.99%)	22,247,146 (52.82%)
GL3	46,840,012	40,790,361 (87.08%)	35,063,843 (74.86%)	5,726,518 (12.23%)	24,414,348 (52.12%)	24,326,796 (51.94%)
YL1	42,372,458	37,396,448 (88.26%)	32,031,262 (75.59%)	5,365,186 (12.66%)	22,396,444 (52.86%)	22,374,279 (52.80%)
YL2	41,179,138	36,322,600 (88.21%)	31,101,043 (75.53%)	5,221,557 (12.68%)	21,737,644 (52.79%)	21,724,824 (52.76%)
YL3	40,957,672	36,016,357 (87.94%)	30,889,952 (75.42%)	5,126,405 (12.52%)	21,530,251 (52.57%)	21,507,102 (52.51%)

Note: Sample: the sample ID in the system; total reads: the counts of clean reads, counted as single-end; mapped reads: counts of mapped reads and their proportion in the clean data; unique mapped reads: counts of reads mapped to a unique position on the reference genome and their proportion in the clean data; multiple mapped reads: counts of reads mapped to multiple positions on the reference genome and their proportion in the clean data; reads map to '+': counts of reads mapped to the sense chain and their proportion in the clean data; reads map to '-': counts of reads mapped to the antisense chain and their proportion in the clean data.

Table S3. Statistical analysis of clean data.

Sample ID	Read Number	Base Number	N50	Mean Length	Max Length	Mean Q-score
GL	6,194,057	6,164,104,419	1,060	995	27,179	Q13
YL	4,305,604	6,074,872,275	1,636	1,410	192,498	Q14

Note: Sample ID: the sample name; read number: the number of sequences; base number: the total base number; N50: the N50 length; mean length: the mean read length; max length: the maximum read length; mean Q-score: the mean quality value.

Table S4. Statistical analysis of full-length sequences.

Sample ID	Number of clean reads (excluding rRNA)	Number of full-length reads	Percentage of full-length reads (FL%)
GL	5,099,579	4,584,518	89.90%
YL	4,060,543	3,645,430	89.78%

Note: Sample ID: name of sample; number of clean reads (except rRNA): number of clean read sequences after filtering rRNA; number of full-length reads: number of full-length sequences; full-length percentage (FL%): percentage of full-length sequences.

Table S5. qPCR primers used to amplify *Camellia sinensis* genes.

Gene	Reference genome No.	Forward primer (5′-3′)	Reverse primer (5′-3′)
ADT1	CSS0048392	CCCAATTGCGAAGCAGTTCC	TATGCAACCTGTGCCGAAGT
ADT1 X1	CSS0023512	GGATTGAGGGATGCTGGTGT	CGGTCGCTTTCTTTGTGGTC
ADT2 X1	CSS0007556	GACGACGAATCACACAACGC	AACCCCCTGGTAAGCAACAC
SHMT	CSS0032127	CACAAGCAGCCAATCCATCG	CGGCAATCTCAGGATCGACA
PSPH	CSS0005049	AACCAGGAGGTGCAGACTTG	TTCGACGACGATGCGATTCT
ASA1	CSS0011355	CCCATTCCACGCCTTGTTTC	GGGACCAAAGAGCCTCCAAT
L-TA	CSS0023615	TGACCAAACCGAGCGAATCA	CCATCTCTGTTTCGAGGCGA
trpB1	CSS0017492	GAACGGCTTACCGAGCACTA	GCTTGAGCCACAGCATTGTT
PHGDH1	CSS0005661	GGGTCGTGTTGGATGGTTCT	TCCAAATGACCCCACCATCG
ADH1	CSS0012905	CATGATTTACACGCGGCTGG	TCCAACTGCTCCATCGCATT
DHAD	CSS0027237	AATCACCGAACCCAAGTCCC	TGAACCCAACCATACCAGCC
BCAT2	CSS0036801	CATCTCCCCTCCAAGAAGCC	TAAGACTCCGGCAGAAGGGT
leuC	CSS0008180	GAGTCCTACTTGCGGTGCTT	TTTGTGTCCCATCCGACCAG
β-actin	TEA019484.1	GAGATTCCGTTGCCCTGA	AGCACAATGTTGCCATAGAGA
ONT.12712.2	PQ821760	GTTGGGTGGAACCGACTATGT	GAAGTATCTTGCCTCGTGGCTT
ONT.7520.1	PQ821761	TGTGCCTCACTTCGAGGTTC	TGGACATAAGCACATCCAACCA
ONT.7520.2	PQ821762	TTCTCGAGCCAAGGCTGTTT	GTGGACATAAGCACATCCAACC
ONT.3025.2	PQ821763	ACCCCACAGCAAGTGAATGG	TCGGCAACAACAGGTGGATT
ONT.14673.1	PQ821764	CGTTGTGGTCTTCGCAATGG	GCACTCGGCCAAACAACAAG
